# Evaluating the effectiveness of trematocides against *Fasciola gigantica* and amphistomes infections in cattle, using faecal egg count reduction tests in Iringa Rural and Arumeru Districts, Tanzania

**DOI:** 10.1186/s13071-018-2965-7

**Published:** 2018-07-03

**Authors:** Jahashi Nzalawahe, Rose Hannah, Ahmed A. Kassuku, John R. Stothard, Gerald Coles, Mark C. Eisler

**Affiliations:** 1 Department of Veterinary Microbiology, Parasitology and Biotechnology, Sokoine University of Agriculture, Morogoro, Tanzania; 2 School of Veterinary Sciences, University of Bristol, Langford House, Langford, Bristol, BS40 5DU UK; 3 Department of Parasitology, Liverpool School of Tropical Medicine, Liverpool, UK

**Keywords:** Cattle, *Fasciola gigantica*, Amphistomes, Trematocide, Efficacy, Tanzania

## Abstract

**Background:**

Fasciolosis, caused by the liver fluke *Fasciola gigantica*, and paramphistomosis are widespread in cattle in Tanzania, and the use of trematocides is encouraged by the Government livestock extension officers. However, reduced efficacy of oxyclozanide against *Fasciola gigantica* and amphistomes (rumen flukes), and albendazole against *F. gigantica*, has been reported in some regions. This study was conducted to assess the efficacy of different trematocides against *F. gigantica* and amphistome infections in cattle at Iringa Rural and Arumeru Districts.

**Methods:**

Cattle found with concurrent infection of *F. gigantica* and amphistomes were randomly grouped into six experimental groups. One control group was left untreated while five treatment groups were treated with one of five trematocides that include: albendazole, nitroxynil, oxyclozanide, closantel and triclabendazole. Post-treatment faecal sample collection was done on the day of treatment and again at 7, 14 and 28 days, from each cattle. The samples were processed by Flukefinder® method to recover and identify eggs. Assessment of the efficacy of the trematocides against *F. gigantica* and amphistomes was conducted using faecal egg count reduction (FECR) tests.

**Results:**

The findings of the present study in both districts indicate that nitroxynil, oxyclozanide, closantel and triclabendazole are effective against patent *F. gigantica* infection, as the calculated FECR% for each trematocide was 100% by day 14 post-treatment. However, albendazole found to have reduced efficacy of against *F. gigantica*, as FECR% was 49% in Arumeru District and 89% in Iringa Rural District by day 14 post-treatment. Oxyclozanide was the only trematocide found to be effective against amphistomes with FECR of 99%.

**Conclusions:**

Albendazole had reduced efficacy against *F. gigantica* in cattle in Arumeru and Iringa Rural Districts, Tanzania. The reduced efficacy was prominent in Arumeru, where cattle are commonly treated with anthelmintics, than in Iringa Rural, where cattle are seldom treated.

## Background

Trematode infections, including fasciolosis and paramphistomosis, are the commonest helminth infections in domesticated ruminants in Tanzania [1]. Fasciolosis, caused by the liver fluke *Fasciola gigantica*, is widespread in the country [2, 3]. In contrast, *F. hepatica* appears to be restricted to the Kitulo plateau in southern highlands zone [4]. Recent surveys of cattle in Tanzania observed monthly prevalences of *F. gigantica* up to 89% in cattle presented for slaughter in western Tanzania [5] and between-herd prevalences of up to 100% in some wards [6]. Fasciolosis can incur a significant economic cost at slaughter [7] but the full economic and social impact of subclinical and chronic infections is unknown, and represents a major public health concern. Scanty information is available on the amphistome species responsible for causing paramphistomosis; however, a previous study [8] reported the presence of *Calicophoron microbothrium* and *Cotylophoron jacksoni* in Iringa.

Control of trematode infections is mainly achieved through application of trematocides. The commonly used flukicides for treatment of *F. hepatica* infection are albendazole, triclabendazole, nitroxynil, closantel, oxyclozanide, rafoxanide and clorsulon, which have different spectrum of activity against flukes of different ages [9]. The salicylanilides appear effective against both *Fasciola* spp. and amphistomes and among these oxyclozanide is the recommended drug for the treatment of amphistomes [10–13]. Tanzanian Government livestock extension officers [14] and Community Animal Health Workers [6] recommend the use of trematocide to treat and control trematode infections and improve cattle productivity. However, trematocide resistance has been reported in many countries worldwide [15–19], including Tanzania; reduced efficacy of oxyclozanide against *F. gigantica* and amphistomes, and albendazole against *F. gigantica*, has been reported on an Amani dairy farm in the Kilolo District, Iringa region [20]. This study was designed to determine the efficacy of the commonly used trematocides in Tanzania that include albendazole, nitroxynil and oxyclozanide against *Fasciola* and amphistome infections in cattle in the Arumeru and Iringa Rural Districts.

## Methods

### Study area and cattle management systems

Cattle involved in this trial were improved dairy breeds (*Bos taurus* × *B. indicus*) in intensive and semi-intensive cattle management systems in the Arumeru District and indigenous breeds (*B. indicus*) in traditional management systems in the Iringa Rural District [21]. The Arumeru District has three agricultural zones based on the altitude and rainfall that include; coffee-banana (upper zone), middle and lower zones. The coffee-banana zone is characterized by coffee-banana inter-cropping and intensive cattle management systems and has the highest annual rainfall (more than 1200 mm). Middle zone has annual rainfall of about 900–1200 mm, with cattle rearing being intensive and semi intensive management systems, whilst the lower zone has the lowest annual rainfall of about 600–800 mm and cattle’s keeping is extensive management system. The study involved the villages from the coffee-banana and middle zones. The livestock farmers in Arumeru District deworm cattle regularly at a range of 2–4 times a year. The Iringa Rural District is an area of high annual rainfall (up to 1600 mm) situated in the southern highlands of Tanzania [21]. The study site was located in a village ~1600 metres above sea level and is characterized by an extensive floodplain that is inundated with water during the rainy season (December to May) and grazed communally by livestock from the adjacent village the remainder of the year. No treatment records were available for the cattle sampled. However, traditionally managed cattle in the region are very rarely treated for parasites [8]. It is therefore likely that the majority of the cattle in this study have never received anthelmintic treatments.

### Experimental design

The study was conducted in August and September 2013 (Arumeru) and September and October 2014 (Iringa). Five days before beginning the trial in each District, faecal samples were collected from 120 cattle and *Fasciola* spp. and amphistome eggs enumerated as described below. Cattle were numbered 1–120 using a marking crayon for later identification. Faecal samples were processed using Flukefinder® (Richard Dixon, ID, USA) and recovered eggs were identified and counted using a stereo microscope [1]. *Fasciola* eggs were distinguished from the amphistome eggs based on their morphological characteristics [22, 23]. Due to the restricted geographical distribution of *F. hepatica* in Tanzania, it is likely that the *Fasciola* eggs observed in this study are of *F. gigantica.* Sixty animals of all ages (excluding suckling calves) with concurrent infection of *F. gigantica* and amphistomes were selected based on faecal egg count. The selected animals were allocated into six experimental groups of 10 animals each, stratified by faecal egg count to ensure that each group included animals with a range of egg counts. On day 0 (day of treatment) five groups were treated with one of five trematocides purchased in the United Kingdom (Table 1) while a control group was left untreated. Triclabendazole and closantel are not commonly available in Tanzania, but were included for comparative purposes. All animals were ear tagged for identification. In addition, animals were marked with a marking crayon and details of sex and coloration noted in case of loss of ear tags. Post-treatment faecal sample collections were done on the day of treatment and again at 7, 14 and 28 days, from each cattle.

**Table 1:** Trematocides used in the trial against *F. gigantica* and amphistome infections in cattle

Trematocide	Trade name	Dose (mg/kg)	Administration route
Albendazole	Albex 10%	7.5	Oral
Nitroxynil	Trodax 34%	10	Subcutaneous
Oxyclozanide	Zanil	10	Oral
Closantel	Flukiver 5%	10	Oral
Triclabendazole	Fasinex 240 24%	12	Oral

### Statistical analysis

Differences in faecal egg counts on day 0 between treatment groups were evaluated using the Kruskal-Wallis test in R [24].

The method for the detection of anthelmintic resistance in nematodes [25] was applied to fluke in this study using the “*egg Counts*” R package [26]. Effectiveness of a trematocide was determined by calculating the faecal egg count reduction (FECR) and was considered effective when the calculated FECR was ≥ 95% and 95% lower confidence limit (LCL) was ≥ 90% [25]. A limitation of FECR is the variable nature of flukes egg shedding in the host faeces [27] and hence faecal egg counts of day 14 post-treatment were used in FECR calculations as recommended by previous studies [15, 18, 27]. Due to potential variability in the accuracy of the faecal egg counting method for fluke [28], percentages of individuals in each group that were positive for infection on day 14 and/or day 28 post-treatment are also presented, where individuals with ≥ 1 egg/g were assumed to be infected (positive), and those with 0 eggs/g were assumed to be uninfected [29].

## Results

In Iringa Rural District, two animals from the triclabendazole group were not returned for repeat sampling and were therefore excluded from analysis. Only 8 individuals were allocated to the closantel group and 9 to the control and albendazole groups due to escape of the remaining assigned individuals from the enclosure at the time of treatment (day 0). No animals were lost in Arumeru. Individual faecal egg counts are provided in Tables 2 and 3.

**Table 2:** Individual egg counts (eggs per gram) and demographic information for cattle treated in the Arumeru District

Animal ID^a^	Sex	Age group	*Fasciola gigantica*				Amphistomes			
			Day 0	Day 7	Day 14	Day 28	Day 0	Day 7	Day 14	Day 28
CO1	Female	Adult	5	1	2	0	17	11	3	9
CO2	Female	Adult	12	4	3	6	16	10	6	15
CO3	Female (pregnant)	Adult	109	4	7	3	726	120	95	40
CO4	Female	Adult	23	3	9	4	34	11	7	19
CO5	Female	Adult	18	4	4	2	137	22	57	37
CO6	Female	Adult	8	3	0	0	12	4	2	4
CO7	Female	Calf	12	2	1	3	71	10	8	30
CO8	Female	Adult	21	5	5	32	7	2	2	5
CO9	Female	Adult	6	0	0	1	28	16	8	6
CO10	Female	Adult	4	2	3	1	121	86	79	138
AL1	Female (pregnant)	Adult	5	1	2	0	17	11	3	9
AL2	Female (pregnant)	Adult	12	4	3	6	16	10	6	15
AL3	Female	Adult	109	4	7	3	728	120	95	40
AL4	Female	Adult	23	3	9	4	34	11	7	19
AL5	Female (pregnant)	Adult	18	4	4	2	137	22	57	37
AL6	Female	adult	7	3	0	0	12	4	2	4
AL7	Female	Adult	12	2	1	3	71	10	8	30
AL8	Female	Calf	21	5	5	32	7	2	2	5
AL9	Female	Adult	6	0	0	1	28	16	8	6
AL10	Female	Adult	3	2	1	1	121	86	79	138
TR1	Female	Calf	14	0	0	0	13	10	6	3
TR2	Female	Adult	6	0	0	0	55	110	16	65
TR3	Female	Adult	7	0	0	0	7	19	20	7
TR4	Female	Adult	33	0	0	0	131	93	45	46
TR5	Female	Adult	3	0	0	0	9	6	1	1
TR6	Female	Heifer	8	0	0	0	10	5	7	7
TR7	Female	Adult	18	0	0	0	4	10	4	6
TR8	Female (pregnant)	Adult	6	0	0	0	11	7	2	6
TR9	Female	Adult	6	0	0	0	22	37	13	13
TR10	Female	Adult	63	0	0	0	6	0	0	3
ZAN1	Female	Adult	4	0	0	0	267	0	2	11
ZAN2	Female	Calf	7	0	0	0	4	1	0	0
ZAN3	Female	Adult	10	0	0	0	4	0	0	0
ZAN4	Female	Adult	17	0	0	0	72	1	1	0
ZAN5	Female	Adult	9	0	0	0	92	0	0	0
ZAN6	Male	Calf	31	0	0	0	45	0	3	7
ZAN7	Female	Adult	15	0	0	0	11	0	0	0
ZAN8	Female	Adult	10	0	0	0	8	0	0	0
ZAN9	Female	Calf	8	0	0	0	2	0	0	0
ZAN10	Female	Adult	4	1	0	0	22	0	3	0
CL1	Female	Calf	11	0	0	0	1	0	0	0
CL2	Male	Adult	20	0	0	0	1	1	33	4
CL3	Female	Adult	7	0	0	0	82	46	0	42
CL4	Female	Adult	5	0	0	0	390	427	316	139
CL5	Male	Calf	7	0	0	0	5	5	0	3
CL6	Male	Adult	44	0	0	0	3	5	1	5
CL7	Female	Adult	12	0	0	0	4	3	1	0
CL8	Male	Calf	16	0	0	0	0	0	0	2
CL9	Female	Adult	26	0	0	0	4	0	0	2
CL10	Female	Adult	7	0	0	0	17	32	19	21
FAS1	Female	Adult	48	0	0	0	3	1	1	0
FAS2	Male	Adult	12	0	0	0	4	1	1	1
FAS3	Female	Adult	34	0	0	0	153	108	36	53
FAS4	Female	Calf	12	0	0	0	1	0	0	0
FAS5	Male	Adult	14	0	0	0	19	19	11	-
FAS6	Female	Adult	5	0	0	0	20	11	12	7
FAS7	Male	Adult	19	0	0	0	7	12	4	4
FAS8	Male	Weaner	4	0	0	0	3	1	2	0
FAS9	Female	Adult	3	0	0	0	33	65	45	11
FAS10	Female	Calf	3	0	0	0	1	0	0	0

^a^Treatment group is indicated in the ID prefix, where CO = control, ALB = Albenil, TR = Trodax, ZAN = Zanil, CL = Closantel (Flukiver) and FAS = Fasinex

**Table 3:** Individual egg counts (eggs per gram) and demographic information for cattle treated in the Iringa Rural District

Animal ID^a^	Sex	Age group	*Fasciola gigantica*				Amphistomes			
			Day 0	Day 7	Day 14	Day 28	Day 0	Day 7	Day 14	Day 28
CO1	Male	Adult	3	8	8	1	18	91	119	311
CO2	Female	Adult	8	4	0	3	79	24	87	32
CO3	Male	Adult	21	14	10	19	679	387	395	556
CO4	Male	Calf	11	6	7	4	260	174	116	479
CO5	Female	Adult	49	42	38	105	16	54	48	10
CO6	Male	Adult	2	8	16	2	46	111	228	90
CO7	Female	Adult	62	56	0	12	262	136	140	326
CO8	Female	Adult	12	64	14	5	21	107	167	92
CO9	Female	Adult	253	97	6	59	16	51	22	8
ALB1	Male	Adult	22	6	0	5	29	13	13	40
ALB2	Female	Adult	16	3	0	0	41	87	74	39
ALB3	Male	Adult	26	3	0	0	375	14	63	177
ALB4	Male	Adult	38	1	8	5	127	67	66	19
ALB5	Male	Adult	12	0	1	0	51	104	171	229
ALB6	Male	Adult	53	0	0	0	244	9	182	298
ALB7	Male	Calf	13	0	1	1	9	2	0	26
ALB8	Female	Adult	37	0	0	0	29	58	149	60
ALB10	Female	Adult	3	0	0	0	97	26	161	235
TR1	Female	Calf	3	0	0	0	17	136	159	218
TR2	Male	Adult	11	3	0	0	575	13	110	299
TR3	Male	Adult	46	0	0	0	0	0	0	0
TR4	Female	Adult	28	0	0	0	170	576	987	268
TR5	Female	Adult	45	0	0	0	429	336	46	241
TR6	Male	Adult	2	0	0	0	39	164	155	377
TR7	Male	Adult	10	0	0	0	430	10	151	498
TR8	Female	Adult	26	0	0	0	4	52	45	38
TR9	Male	Calf	2	0	0	0	6	0	9	50
TR10	Male	Adult	21	0	0	0	24	44	23	17
ZAN1	Male	Adult	7	0	0	0	6	0	0	1
ZAN2	Female	Adult	144	5	0	0	93	1	0	0
ZAN3	Male	Adult	24	0	0	0	211	0	0	0
ZAN4	Male	Adult	28	3	0	0	9	0	0	0
ZAN5	Male	Adult	112	0	0	0	133	0	0	2
ZAN6	Female	Adult	33	0	0	0	84	0	0	0
ZAN7	Female	Adult	94	0	0	0	13	0	5	0
ZAN8	Female	Adult	48	0	0	0	48	0	0	0
ZAN9	Male	Adult	18	0	0	0	220	0	11	43
ZAN10	Female	Adult	253	2	0	0	22	1	0	0
CL1	Female	Adult	18	0	0	0	41	28	2	17
CL2	Female	Adult	21	0	0	0	106	7	92	95
CL3	Female	Adult	8	1	0	0	208	477	24	363
CL4	Female	Adult	39	3	0	0	53	1	44	19
CL5	Male	Adult	2	0	0	0	87	31	6	41
CL6	Female	Adult	49	0	0	0	51	9	19	18
CL7	Male	Adult	3	0	0	0	131	150	143	106
CL8	Female	Adult	16	0	0	0	5	3	0	0
FAS1	Male	Adult	12	0	0	0	2	0	7	4
FAS4	Female	Adult	15	0	0	0	63	46	12	23
FAS5	Male	Adult	241	0	0	2	110	109	157	187
FAS6	Male	Adult	8	-	0	0	77		234	254
FAS7		Adult	27	5	0	0	39	30	18	65
FAS8	Female	Adult	50	1	0	0	10	16	60	8
FAS9	Male	Adult	12	0	0	0	0	7	1	0
FAS10	Male	Adult	12	0	0	0	4	0	5	18

^a^Treatment group is indicated in the ID prefix, where CO = control, ALB = Albenil, TR = Trodax, ZAN = Zanil, CL = Closantel (Flukiver) and FAS = Fasinex

Mean (± SD) *F. gigantica* and amphistomes faecal egg counts for the study animals in Arumeru on day 0 were 16.78 ± 20.87 and 64.60 ± 141.58, respectively. There were no significant differences in *F. gigantica* faecal egg counts between treatment groups on day 0 (*χ*^2^ = 0.72, *df* = 5, *P* = 0.98). There was a significant difference in amphistomes faecal egg counts between treatment groups (*χ*^2^ = 13.09, *df* = 5, *P* = 0.02), but this was due to three individuals with high faecal egg counts in the control, albendazole and nitroxynil groups.

Mean (± SD) *F. gigantica* and amphistomes faecal egg counts for the study animals in Iringa on day 0 were 39.43 ± 58.12 and 109.61 ± 147.95, respectively. There were no significant differences in faecal egg counts between treatment groups on day 0 (*F. gigantica: χ*^2^ = 7.15, *df* = 5, *P* = 0.21; amphistomes: *χ*^2^ = 3.90, *df* = 5, *P* = 0.56).

Nitroxynil, oxyclozanide, closantel and triclabendazole were highly effective against patent *F*. *gigantica* infections in cattle, reducing faecal egg counts and number of infected animals by 100%, with the exception of one individual in the triclabendazole group in Iringa that produced 2 eggs/g of faeces on day 28 (Tables 3, 4). Reduced efficacy of albendazole was observed in both Iringa and Arumeru Districts and was more pronounced in the Arumeru District (Tables 2, 4). The decrease in faecal egg count in both districts was greater than the decrease in number of infected animals. In Arumeru 90% of animals treated with albendazole were observed to be positive for *F. gigantica* infection on day14 and/or 28 despite a 50% faecal egg count reduction. In Iringa Rural District, 44% of individuals remained positive, despite a 90% faecal egg count reduction on day 14 post-treatment (Table 4). Oxyclozanide was the only flukicide found to be effective against patent amphistome infections in cattle (Table 2).

**Table 4:** Efficacies of trematocides to *F. gigantica* and amphistomes in infected cattle and percentage positive on day 14 and/or day 28 post-treatment

Trematocide	*Fasciola gigantica*			Amphistomes			District
	FECR (%)	95% LCL	% positive (*n*)	FECR (%)	95% LCL	% positive (*n*)	
Albendazole	50	0	90 (9/10)	50	0	100 (10/10)	Arumeru
	90	38	44 (4/9)	34	0	100 (9/9)	Iringa Rural
Nitroxynil	100	100	0 (0/10)	79	33	100 (10/10)	Arumeru
	100	100	0 (0/10)	-15	0	90 (1/10)	Iringa Rural
Oxyclozanide	100	100	0 (0/10)	99	95	40 (4/10)	Arumeru
	100	100	0 (0/10)	99	95	40 (4/10)	Iringa Rural
Closantel	100	100	0 (0/10)	31	0	50 (5/10)	Arumeru
	100	100	0 (0/8)	72	18	88 (7/8)	Iringa Rural
Triclabendazole	100	100	0 (0/10)	79	25	80 (8/10)	Arumeru
	100	100	13 (1/8)	58	0	100 (8/8)	Iringa Rural
Control	na	na	90 (9/10)	na	na	100 (10/10)	Arumeru
	na	na	100 (9/9)	na	na	100 (9/9)	Iringa Rural

*Abbreviations: FECR* faecal egg count reduction, *LCL* lower confidence limit

## Discussion

The results of the current work clearly show that nitroxynil, oxyclozanide, closantel and triclabendazole are highly effective against *F. gigantica* in naturally infected Zebu cattle and improved Zebu breeds. These findings concur with previous studies in Tanzania that reported 100% efficacy of nitroxynil and triclabendazole [20] and 96.7–100% efficacy of oxyclozanide [30] against *F. gigantica* in naturally infected cattle*.* Comparable results based on the faecal egg count reduction test and controlled anthelmintic trials in cattle, sheep and goats have been reported elsewhere in the world [17–19, 31–38]. However, an apparent reduced efficacy of albendazole against *F. gigantica* was observed in this study. Equivalent findings on treatment failure of albendazole to *F. gigantica* have been reported elsewhere in Tanzania [20]. Reduced efficacy of albendazole against *F. gigantica* in the present study can be explained by the widespread use of albendazole in Tanzania for control of nematode infections in domesticated ruminants. Mixed infections of trematodes and gastrointestinal nematodes are very common in cattle, general practices of livestock field officers/farmers in the study areas is mainly to use clinical signs for diagnosis of helminth infections and when albendazole is used to treat suspected nematode infections in cattle adult *F. gigantica* will be exposed to the lower dose of 7.5 mg/kg, which is recommended for nematodes, instead of 10 mg/kg as recommended for adult liver flukes. Underdosing is known to be a factor that can lead to selection for resistance in nematodes [5] and this could explain the reduced efficacy of albendazole to *Fasciola*.

The albendazole FECR of 49% in the Arumeru District was much lower compared to the Iringa Rural District (89%), which could be attributed to the routine deworming (2–4 doses per annum) practices in small scale dairy farmers in Arumeru District compared to the traditional livestock farmers in Iringa Rural District that deworm animals when they are clinically sick. However, it is likely that the majority of cattle treated in Iringa had never been exposed to albendazole, and although the impact of historic treatments on the contamination of pasture by resistant fluke cannot be ruled out, it was considered highly unlikely and therefore the reduced efficacy of this drug in Iringa was a surprise.

Albendazole is only effective against adult *Fasciola hepatica* [9, 33]. Assuming a similar situation in *F. gigantica*, immature fluke present at the time of treatment may have matured within the study period and continued to shed eggs, resulting in the observed treatment failure of albendazole. However the 100% efficacy of oxyclozanide which is also only effective against adult *F. hepatica* [9] indicates a reduced efficacy of albendazole against *F. gigantica* in these cattle for other reasons than its mode of action.

Cattle were generally in poor body condition and suffered from multiple parasitic infections in addition to liver fluke, including *Schistosoma bovis*, trichostrongylid nematodes, ticks and mite infestations. It is therefore unlikely that individual weights were underestimated by using the weight tape and therefore under-dosing is unlikely. Cattle were not fasted prior to treatment and evidence of decreased gut transit time (grains in faeces) and subclinical acidosis (sour smelling faeces) was observed in cattle in the Iringa district, which may affect the pharmacokinetics of albendazole [39]. However, if this were the case, then a similar impact on other orally administered trematocides would be expected.

The reduced efficacy of albendazole might be due to features of the Zebu cattle compared with European breeds (e.g. reduced digestive retention times [40] or differences in the pharmacokinetic profile of some drugs [41]). Products containing albendazole are licensed for the control of *F. hepatica* in cattle and therefore the reduced efficacy could also be due to fundamental differences between the susceptibility of *F. hepatica* and *F. gigantica* to the drug.

Oxyclozanide was the only drug found to be effective against amphistomes, in agreement with previously reported findings [12, 13, 42, 43]. However, the observed inefficacy of closantel (10 mg/kg) against amphistomes in this study is contrary to the findings of Arias et al. [10] in Spain who found closantel to be effective against the amphistome *Calicophoron daubneyi*. Since the current work did not characterize the amphistomes to the species level, the species in the study areas could be other than *Calicophoron daubneyi*.

Flanagan et al. [27] recommends that efficacy of a trematocide should be determined by FEC of day 14 post-treatment in domesticated ruminants infected with *F. hepatica*, as this sampling time allows the clearance of stored *Fasciola* eggs from the host gall bladder. However this study has observed that in naturally infected cattle with *F. gigantica* that were treated with the effective trematocides FEC levels fell to negative values within seven days after treatment at Arumeru District, with some cattle having low counts at Iringa Rural District. Similar findings have been observed by Brockwell et al. [15], who reported FEC were reduced to zero by day 7 post-treatment in cattle infected with *F. hepatica* after being treated with the effective trematocide.

## Conclusions

Nitroxynil, oxyclozanide, closantel and triclabendazole were highly effective against patent *F. gigantica* infection in naturally infected cattle. Oxyclozanide was also found to be highly effective against patent paramphistome infection. Treatment failure of albendazole against *F. gigantica* was observed in both Districts*.* In the Arumeru District, where albendazole is used regularly to control helminth infections, anthelmintic resistance in *F. gigantica* may contribute to the observed treatment failure, as lower FECR were observed in Arumeru than Iringa Rural. However, in Iringa Rural District, where there is very little history of chemotherapeutic treatment of the cattle, the reason for treatment failure is unknown. However, as a baseline efficacy of albendazole against *F. gigantica* in *B. indicus* and *B. indicus* × *B. taurus* has not, to the authors’ knowledge, been established, the possibility of reduced efficacy of albendazole against *F. gigantica* warrants further investigations.

## Abbreviations

*df*: Degrees of freedom
FECR: Faecal egg count reduction test
LCL: Lower confidence limit
SD: Standard deviation
*χ*^2^: Chi-square test
